# Rodlike YMn_2_O_5_ Powders Derived from Hydrothermal Process Using Oxygen as Oxidant

**DOI:** 10.3390/ma13030805

**Published:** 2020-02-10

**Authors:** Jun Shi, Jing Wang, Huifen He, Yang Lu, Zhongxiang Shi

**Affiliations:** 1 Liaoning Key Laboratory for Fabrication and Application of Superfine Inorganic Powders, Dalian Jiaotong University, Dalian 110621, China; shijun@djtu.edu.cn (J.S.); hehuifen163@163.com (H.H.); luyang4967@126.com (Y.L.); szx492670794@163.com (Z.S.); 2 Department of Materials Science and Engineering, Yingkou Institute of Technology, Yingkou 115014, China

**Keywords:** yttrium manganite, hydrothermal method, formation mechanism, luminescent properties

## Abstract

A facile approach is proposed herein to fabricate YMn_2_O_5_ powders with the hydrothermal method with oxygen as an oxidant. The structure and morphology of the as-synthesized YMn_2_O_5_ powders were characterized by XRD, SEM, and high-resolution transmission electron microscopy (HRTEM). The results manifested that the main factors that affected the formation of the rod-like YMn_2_O_5_ structures were the stirring time, hydrothermal temperature, and hydrothermal time. The oxidation time in the air had a remarkable effect on the final product by oxidizing Mn^2+^ ions to Mn^3+^ ions and Mn^4+^ ions. The obtained YMn_2_O_5_ powder was single crystalline and possessed a nanorod morphology, where the growth direction was along the c axis. The possible formation mechanism involved a dissolution–crystallization mechanism. Under the 397 nm excitation, the Mn^4+^ ions exhibited an intense orange emission at 596 nm. The energy bandgap of YMn_2_O_5_ powders was 1.18 eV.

## 1. Introduction

Multiferroic materials, which are a kind of multifunctional material with two or more kinds of ferroelectric and ferromagnetic properties, have been widely applied in the fields of material science and condensed matter physics [1,2,3,4]. In recent years, this kind of material has attracted extensive attention and resulted in the development of new magnetoelectric devices, spin electronic devices, and high-performance information storage. 

Common perovskite manganese compounds with orthogonal structures are a kind of multiferroic material, such as TbMnO_3_, DyMnO_3_, and TbMn_2_O_5_ [5,6,7,8,9,10]. The conventional approaches to the preparation of perovskite manganese compounds include the solid-state method [11], sol-gel method [12,13], polyacrylamide gel method [14,15,16,17], spark plasma sintering method [18], electrospinning method [19], and hydrothermal method [20,21]. The sol-gel method and the spark plasma sintering method require high-temperature calcination in the intermediate and final stages of synthesis, which may cause product agglomeration and make it difficult to control the reaction. Although the synthesis temperature required for the polymer gel method is high, the purity of the product is low. By comparison, the hydrothermal method is able to control particle size, morphology, and phase and is the most common method for preparing multivalent manganese oxides [20,21]. Therefore, the preparation of nanopowder materials by this method has broad development possibilities. Mn^4+^ ions are usually found on octahedral sites of solids. They exhibit both broadband excitation and sharp emission lines due to the distinct electronic structure [22]. However, the luminescent properties of YMn_2_O_5_ have hardly been reported.

Many pieces of the literature have reported the effect of pH on the morphology of YMn_2_O_5_ powder, and it has been found that nanorod-like powders can be obtained at low pH [21]. In this paper, the effects of air oxidation and hydrothermal conditions on the formation of YMn_2_O_5_ powder were studied, and the formation mechanism of the powder was also discussed. At the same time, the luminescent properties of YMn_2_O_5_ powder were investigated.

## 2. Materials and Methods 

### 2.1. Materials Synthesis

The raw materials included yttrium nitrate hexahydrate (Y(NO_3_)_3_·6H_2_O, Energy Chemical, Shanghai, China), manganese chloride tetrahydrate (MnCl_2_·4H_2_O, DaMao Chemical Reagent Factory, Tianjin, China), and potassium hydroxide (KOH, DaMao Chemical Reagent Factory, Tianjin, China). All chemicals were analytical grade and used without further purification. Certain amounts of MnCl_2_ and Y(NO_3_)_3_ were used to prepare 0.2 and 0.4 mol/L solutions. First, a 4 mmol MnCl_2_ solution and a 9 mmol KOH solution were added to deionized water and stirred in the air at room temperature for different times (0, 10, 15, 20, and 30 min). Then, 10 mL of a Y(NO_3_)_3_ solution was added. Second, the whole solution was transferred into a 100 mL Teflon-lined stainless steel autoclave (HeNan JingHua Instrument Co., ltd, Gongyi, China) with 50% of the volume filled and treated at different temperatures for different times. Finally, the as-synthesized sample was centrifuged and washed with deionized water several times to remove soluble salts and then dried at 60 °C for 12 h. 

### 2.2. Characterization

The crystalline phases of the specimen were analyzed by an Empyrean X-ray diffractometer (PANalytical B.V., Almelo, the Netherlands) with Cu Kα radiation (λ = 0.154056 nm) at a scanning rate of 0.05 deg/s from 10° to 80°. The morphology of the crystals and the particle size were investigated by the ZEISS SUPRA 55 field-emission scanning electron microscope (Carl Zeiss NTS GmbH, Aalen, Germany). The microstructure was characterized by TEM and high-resolution transmission electron microscopy (HRTEM; JEOL 2100F, JEOL Co., Ltd., Tokyo, Japan). Emission and excitation spectra curves were recorded on the F-7000 FL spectrophotometer (HITACHI, Tokyo, Japan). The optical absorption spectra were recorded at a wavelength range of 200–1000 nm by the UV–VIS U-3900 spectrophotometer (HITACHI, Tokyo, Japan).

## 3. Results and Discussion

### 3.1. Effects of Oxidation Time

**Figure 1:**
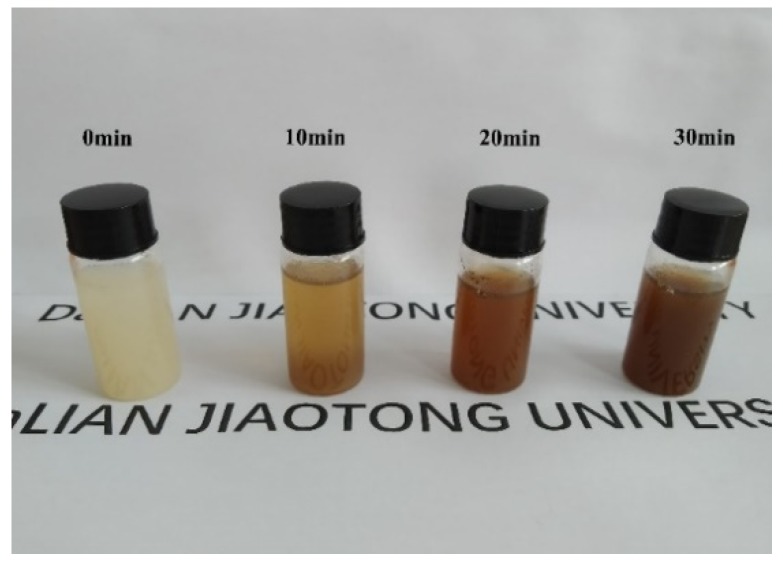
Digital images of the precursor solution with different stirring times.

To study the effect of oxidation time, the colors of the precursor solution in the air for different times were observed, as shown in Figure 1. As the stirring time increased, the color of the precursor solution gradually became darker. This indicated that low-valence manganese ions were gradually oxidized to high-valence manganese by oxygen.

**Figure 2:**
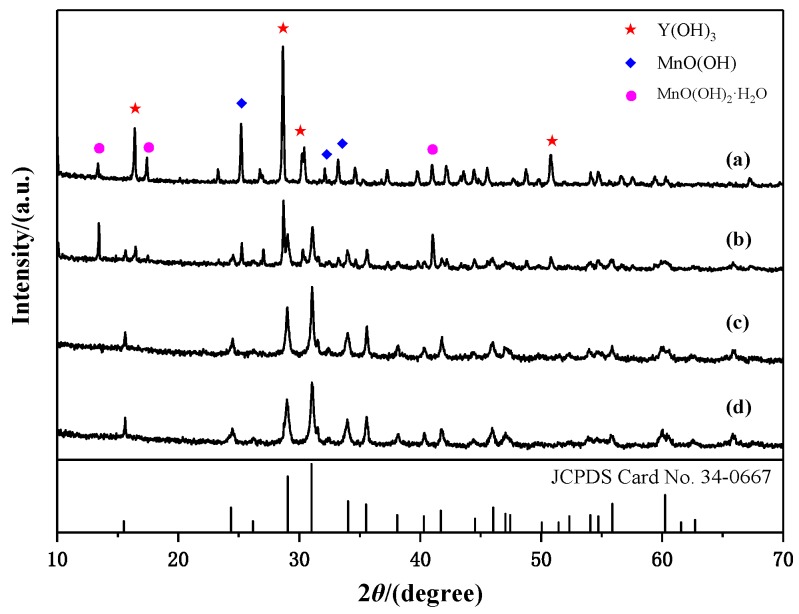
XRD patterns of products obtained by hydrothermal treatment at 180 °C at different oxidation times in the air: (**a**) 0 min; (**b**) 10 min; (**c**) 20 min; (**d**) 30 min.

Figure 2 shows the XRD patterns of hydrothermal products obtained at 180 °C for 24 h after oxidation at different times in the air. Characteristic peaks of Y(OH)_3_, MnO(OH), and MnO(OH)_2_·H_2_O can be seen in Figure 2a. The appearance of high-valence manganese is due to the presence of the air in a confined space during the hydrothermal treatment and also drying in the air. The oxygen present can act as an oxidant, as shown in the following reaction equation [23,24,25,26]:

(1) $$O_{2}+2{H_{2}O+4e}^{-}\to 4{\mathrm{OH}}^{-}.$$

YMn_2_O_5_ in the product was detected, when the stirring time was 10 min, as shown in Figure 2b. Under alkaline conditions, MnCl_2_ reacted with the alkali to form the Mn(OH)_2_ precursor. The reaction equation was as follows:

(2) $${\mathrm{Mn}}^{2+}{+2\mathrm{OH}}^{-}\to {\;\mathrm{Mn}(\mathrm{OH})}_{2}.$$

Mn^2+^ ion and Mn(OH)_2_ were partially or completely oxidized to manganese with a high valence state. The oxidation reaction was favored by increased alkalinity:

(3) $$4{\mathrm{MnCl}}_{2}+8\mathrm{KOH}+O_{2}\to \;4\mathrm{MnO}(\mathrm{OH})+8\mathrm{KCl}+2H_{2}O,$$

(4) $$4\mathrm{MnO}(\mathrm{OH}){+O}_{2}+2H_{2}O\to 4\mathrm{MnO}{\left(\mathrm{OH}\right)}_{2}.$$

When the stirring time increased to 20 min, diffraction peaks from impurity phases disappeared. The main diffraction peaks corresponded to YMn_2_O_5_ (JCPDS Card No. 34-0667). It can be observed that the intensities of the peaks did not change as the stirring time increased.

**Figure 3:**
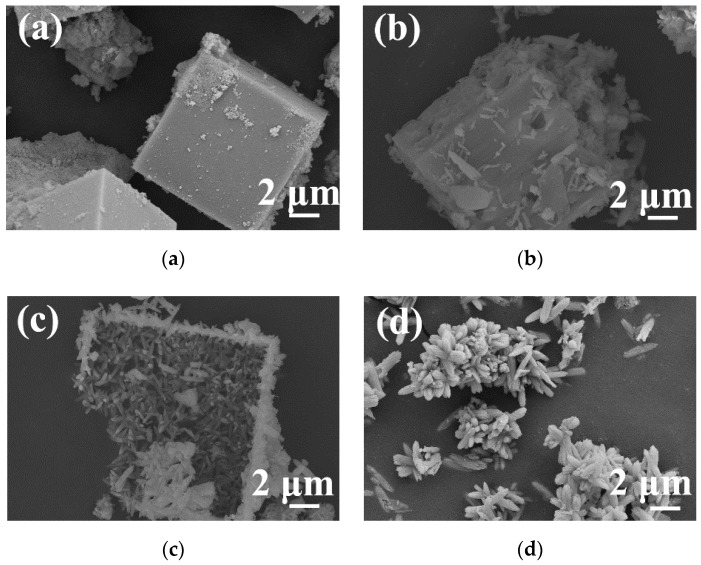
SEM images of hydrothermal products obtained with different stirring times: (**a**) 0 min; (**b**) 10 min; (**c**) 20 min; (**d**) 30 min.

Figure 3 shows the SEM images of hydrothermal products obtained at 180 °C for 24 h after different stirring times. As shown in Figure 3a, when the stirring time was very short, the obtained sample had a solid cubic shape. When the stirring time was 10 min (Figure 3b), the sample underwent a dissolution process, which was indicated by the presence of pores on the surface. When the stirring time was 20 min, a large number of nanoparticles developed into nanorods with an approximately uniform size, as shown in Figure 3c. Upon prolonging the stirring time to 30 min (Figure 3d), the sample became a spindle-like rod that exhibited flower-like aggregates.

### 3.2. Effects of Hydrothermal Conditions

**Figure 4:**
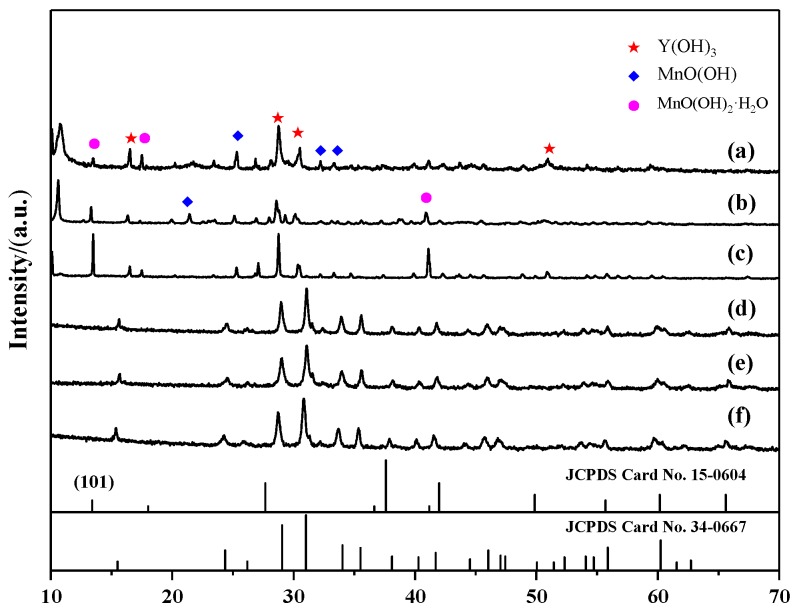
XRD patterns of samples synthesized at different hydrothermal temperatures: (**a**) 140 °C; (**b**) 160 °C; (**c**) 170 °C; (**d**) 180 °C; (**e**) 200 °C; (**f**) 220 °C.

To ascertain the effect of the hydrothermal temperature on the products, hydrothermal synthesis experiments were carried out at six different temperatures for 24 h after stirring for 30 min in the air. The as-prepared samples were analyzed with XRD, as shown in Figure 4. When the hydrothermal temperature increased from 140 to 170 °C, the product was confirmed to be a mixture of Y(OH)_3_, MnO(OH), and MnO(OH)_2_·H_2_O phases, of which the intensity gradually increased, as shown in Figure 4a–c. The crystallite sizes were calculated using the Scherrer equation, i.e., *D* = *K*λ/*B*cosθ. The calculated average crystallite sizes of the (101) crystal plane were 64.5, 77.9, and 87.6 nm, respectively. It was revealed that synthesis temperatures of 170 °C and below were beneficial to the formation of MnO(OH)_2_·H_2_O. When the hydrothermal temperature increased to 180 °C, the XRD results indicated that single-phase YMn_2_O_5_ (JCPDS Card No. 34-0667) was formed successfully, as shown in Figure 4e. The characteristic peaks at 2θ = 15.5°, 24.36°, 29.06°, 31.02°, 34.03°, 35.52°, and 60.24° can be assigned to the corresponding (001), (200), (201), (211), (102), and (112) planes, respectively. No peaks from any other phases were detected, which indicated that the product obtained in the present synthesis condition was highly pure. With the increasing hydrothermal temperature, the XRD diffraction peaks appeared to exhibit no obvious changes, indicating that increasing the temperature further had no effect on the structure of the obtained products. To our knowledge, the formation of the products should overcome the reaction barrier, which can usually be solved by raising the reaction temperature. As mentioned above, the lowest temperature for the synthesis of YMn_2_O_5_ crystallites was 180 °C. Compared with that in Reference [21], the minimum synthesis temperature was reduced by 10 degrees.

**Figure 5:**
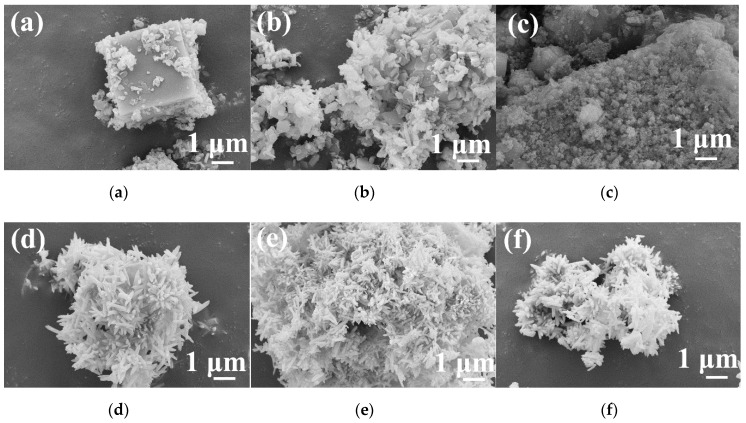
SEM images of samples synthesized at different hydrothermal temperatures: (**a**) 140 °C; (**b**) 160 °C; (**c**) 170 °C; (**d**) 180 °C; (**e**) 200 °C; (**f**) 220 °C.

Figure 5 displays the SEM images of samples prepared at different hydrothermal temperatures for 24 h after stirring for 30 min in the air. The morphology of the sample at 140 °C was regular microcubes with irregular flake-like particles attached to the surface, as presented in Figure 5a. When the hydrothermal temperature increased to 160 and 170 °C, more flake-like particles appeared around irregular microcubes, as shown in Figure 5b,c. When the hydrothermal temperature reached 180 °C, the observed aggregates were composed of microsized rod-like particles, as shown in Figure 5d. Upon elevating the hydrothermal temperature further, there was no apparent change in the morphology. According to Figure 4 and Figure 5, these SEM images demonstrated that the morphology of the samples was related to the crystalline phase of the particles. Obviously, the MnO(OH) and MnO(OH)_2_·H_2_O particles assumed a flake-like shape, and the Y(OH)_3_ particles had a cube-like shape, while the YMn_2_O_5_ nanoparticles took on a rod-like shape.

**Figure 6:**
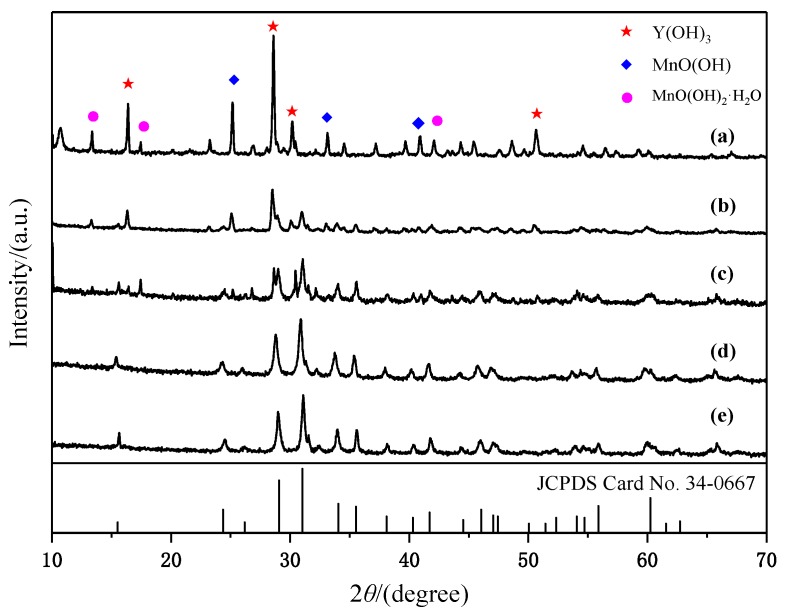
XRD patterns of samples synthesized at 180 °C for different hydrothermal times: (**a**) 6 h; (**b**) 12 h; (**c**) 18 h; (**d**) 24 h; (**e**) 48 h.

Different hydrothermal times were investigated to validate the impact of this parameter. The XRD patterns were obtained after hydrothermal treatments at 180 °C for different times, as shown in Figure 6. When the hydrothermal time was 6 h, MnO(OH), MnO(OH)_2_·H_2_O, and Y(OH)_3_ appeared in the hydrothermal sample. After 12 h, two new diffraction peaks appeared at 2θ values of approximately 29.3° and 30.9°, which coincided with the peaks in JCPDS Card No. 34-0667. This indicated that the YMn_2_O_5_ phase started to form or develop. After 18 h, the intensity of the impurity phase diffraction peaks decreased, while the intensity of the YMn_2_O_5_ peaks increased. After 24 h, the impurity peaks disappeared entirely, and only the diffraction peak from YMn_2_O_5_ remained. At this point, the hydrothermal sample comprised a pure YMn_2_O_5_ powder. There was no apparent effect on the phase structure, after the hydrothermal time increased to 48 h.

**Figure 7:**
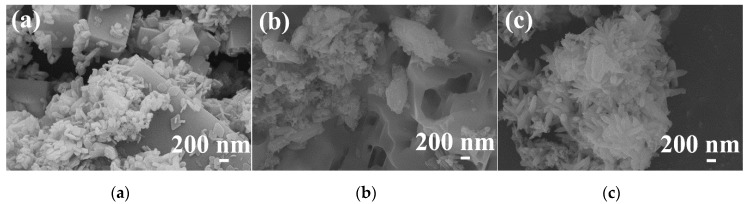

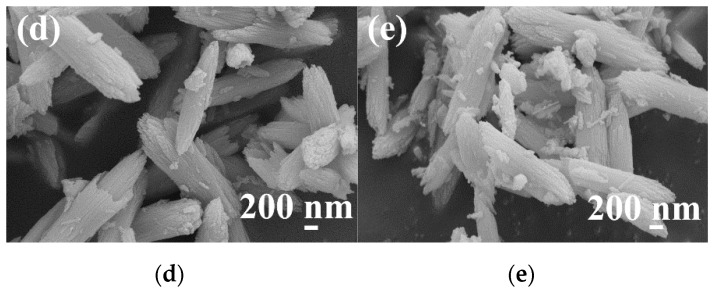
SEM images of samples synthesized at different hydrothermal times: (**a**) 6 h; (**b**) 12 h; (**c**) 18 h; (**d**) 24 h; (**e**) 48 h.

The morphological evolution provides an understanding of the formation process of YMn_2_O_5_ crystals. For this purpose, samples with different hydrothermal times were observed with SEM, as shown in Figure 7. When the hydrothermal time was 6 h, the sample contained cube-like shapes that were covered with flake-like particles. After 12 h, the cube structure began to dissolve, and there were pits and pores that appeared on the surface of the cubes. A small amount of YMn_2_O_5_ seeds formed at this time. When the hydrothermal time increased to 18 h, the flake-like particles disappeared, and rod-like structures appeared. When the hydrothermal time reached 24 h, part of the product morphology appeared spindle-like. Due to the elongation of the holding time, the nucleation position and growth direction of the YMn_2_O_5_ particles had a preferred orientation, which led to the formation of the morphology. When the hydrothermal time extended to 48 h, there was almost no change in the morphology of the product, which is consistent with the results shown in Figure 6.

**Figure 8:**
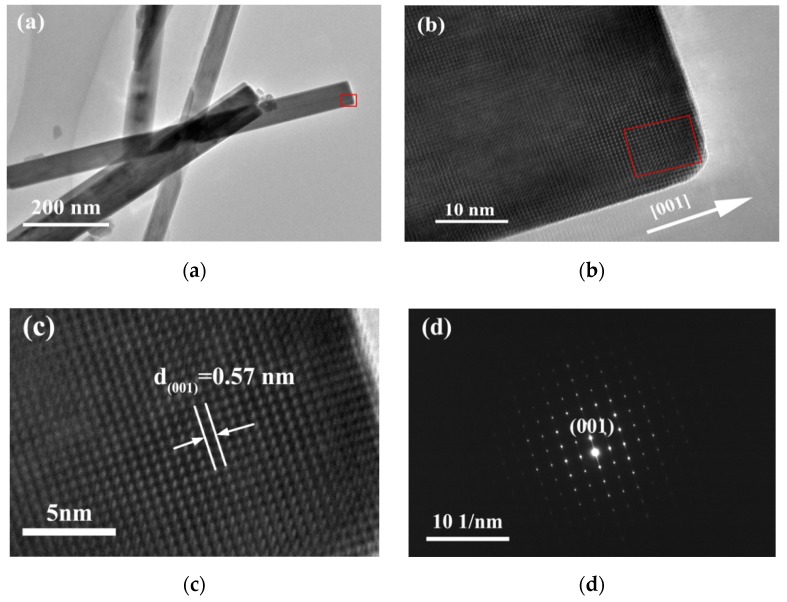
TEM, high-resolution transmission electron microscopy (HRTEM), and selected area electron diffraction (SAED) images of YMn_2_O_5_: (**a**) TEM; (**b**) HRTEM; (**c**) amplification of the HRTEM; (**d**) SAED.

To obtain additional information about the YMn_2_O_5_ powder, TEM, HRTEM, and selected area electron diffraction (SAED) analyses were performed, and the results are shown in Figure 8. Figure 8a further demonstrates that the YMn_2_O_5_ product consisted of a uniform rod-like powder with a diameter range of 50–70 nm. The HRTEM image (Figure 8b,c) clearly shows lattice fringes with an interplanar spacing of 0.57 nm, which is attributed to the (001) plane. Figure 8d shows the SAED pattern of individual nanorods, and the result suggested that the individual YMn_2_O_5_ nanorods were single crystals. According to the above analysis, the growth direction of the YMn_2_O_5_ nanorods was along the [001] direction.

### 3.3. Mechanism Analysis

Based on the above discussion, the formation process and growth mechanism of YMn_2_O_5_ were speculated as following:

(5) $${4Y(\mathrm{NO}}_{3}{)}_{3}{+8\mathrm{MnCl}}_{2}+28\mathrm{KOH}+3O_{2}\to {4\mathrm{YMn}}_{2}O_{5}{+16\mathrm{KCl}+\;12\mathrm{KNO}}_{3}{+14H}_{2}O.$$

Before the hydrothermal reaction, KOH solids were added to the MnCl_2_ solution to obtain Mn(OH)_2_, which can be oxidized into MnO(OH) and MnO(OH)_2_·H_2_O brown precipitates in the air by oxygen [27]. After adding the Y(NO_3_)_3_ solution, a gel was obtained. The precursor solution contained Y^3+^, Mn^3+^, Mn^4+^, and other ions. When the mixture was transferred into the autoclave, the precursor transformed into Y(OH)_x_^3−x^ and Mn(OH)_y_^2x−y^ ion clusters at high temperature and high pressure [28]. As the temperature increased and the time increased, YMn_2_O_5_ compounds were formed continuously by satisfying thermodynamic requirements. Combined with the previously mentioned reaction Equations (1)–(4), the reaction equation was as follows:

(6) $${\mathrm{MnO}(\mathrm{OH})+\mathrm{MnO}(\mathrm{OH})}_{2}{\cdot H}_{2}{O+Y(\mathrm{OH})}_{3}\to {\;\mathrm{YMn}}_{2}O_{5}{+\;4H}_{2}O.$$

According to the classical crystal growth theory, the growth of YMn_2_O_5_ crystals synthesized by the hydrothermal synthesis method can be divided into the following stages. The first is the dissolution stage. Under alkaline conditions, a mixture composed of MnO(OH), MnO(OH)_2_·H_2_O, and Y(OH)_3_ was observed at room temperature. In hydrothermal conditions, precursors were dissolved at high temperature and high pressure and entered into the mixed aqueous solution in the form of Y, Mn ions or ionic groups. Second, there was a thermal convection and concentration difference between the dissolving zone and the growth zone. These ions or clusters reached critical nucleation concentrations that were transported to the growth zone for nucleation. After that, these ions or groups of ions were adsorbed, decomposed, and desorbed at the growth interface, and the adsorbed material moved at the interface. The final step is crystallization [29].

**Figure 9:**
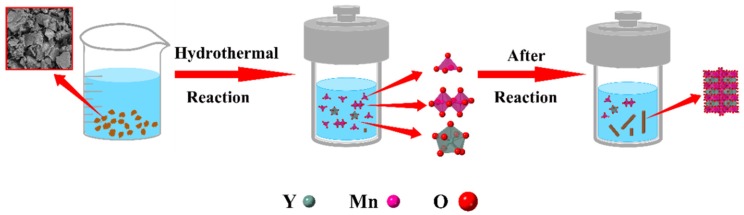
Schematic model of dissolution and recrystallization for YMn_2_O_5_.

The growth mechanism of YMn_2_O_5_ is due to a dissolution–recrystallization mechanism. The schematic in Figure 9 illustrates the process for YMn_2_O_5_ crystals. The insoluble precursors were obtained at room temperature. During the hydrothermal reaction, the precursors, which did not dissolve readily at room temperature, began to dissolve. The ions were attached to the surface of the precursor, which inhibited the overdissolution of the precursor. As the concentration of ions increased, the precursor solution reached a critical nucleation threshold, and YMn_2_O_5_ was obtained by homogeneous nucleation and growth on the surface of the precursor. As the reaction proceeded, the concentration in the system decreased, which promoted the dissolution of the precursor and provided the growth unit for the crystal nucleus. The preferred growth orientation of YMn_2_O_5_ is anisotropic in nature [21,30]. Finally, the growth direction of the crystallized YMn_2_O_5_ was along the crystallography c axis and parallel to the [001] direction.

It is well-known that the product can be influenced by the reaction temperature, time, and concentration. In this work, we paid special attention to the influence of oxygen. Oxygen as an oxidant transformed Mn^2+^ ions into Mn^3+^ and Mn^4+^ ions. The oxidation time is a very critical factor and can affect the balance of manganese ion concentrations in the solution. In addition, sufficient reaction temperature and time should be used to overcome the reaction barrier.

### 3.4. Luminescent Properties

**Figure 10:**
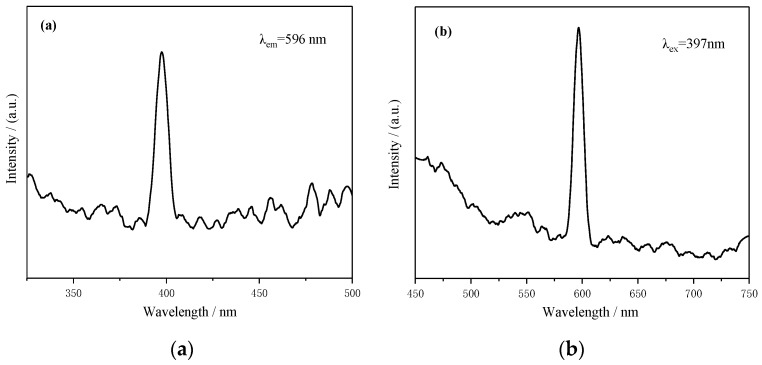
Excitation spectra (**a**) and emission spectra (**b**) of the YMn_2_O_5_ sample.

To investigate the luminescence characteristics of the samples, the excitation and emission spectra were measured and are shown in Figure 10. The sample was prepared at 180 °C for 24 h after stirring for 30 min in the air. All the excitation spectra monitored at 596 nm (see Figure 10a) were composed of a group of sharp lines in the range of 350–500 nm. The strongest excitation peak was located at 397 nm. With the excitation of 397 nm radiation, it can be seen that an intense emission peak was located at 596 nm, as shown in Figure 10b. As well-known, Mn^4+^ ions are in the octahedral crystal field, and orange emission occurs under excitation conditions, which is due to the ^2^E_g_→^4^A_2g_ transition of the 3d orbital energy levels [22]. Due to the Jahn–Teller effect in YMn_2_O_5_ crystals, the lattice distortion of MnO_6_ octahedron caused the blueshift of the Mn emission wavelength [31].

**Figure 11:**
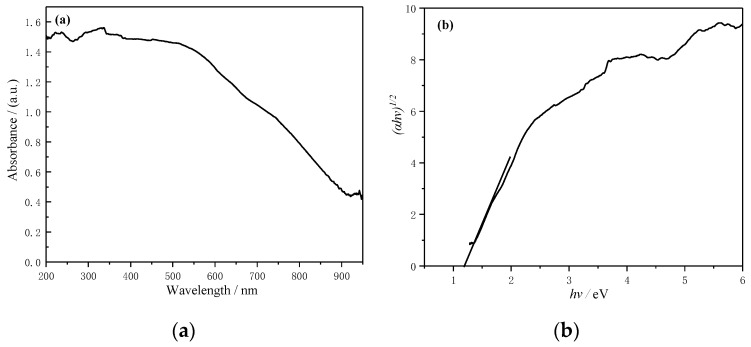
(**a**) UV–VIS absorption spectra of the YMn_2_O_5_ sample, (**b**) Tauc–Mott plot of (αhν)^1/2^ versus hν.

Figure 11a shows the absorption spectra of the YMn_2_O_5_ sample. An absorbance also occurred close to 900 nm, suggesting that the sample can be effectively absorbed in the visible light range. According to the Kubelka–Munk (K–M) theory [32], the indirect bandgap of the YMn_2_O_5_ sample can be estimated from the plot of (αhν)^1/2^ versus hν, as shown in Figure 11b, where α is the K–M absorption coefficient and hν is the incident photon energy. The linear portion of the plot was extrapolated to the axis of the abscissa to yield the energy bandgap. The obtained energy bandgap of the resulted samples was 1.18 eV, which is consistent with Reference [14].

## 4. Conclusions

A hydrothermal method was employed to fabricate YMn_2_O_5_ with oxygen as an oxidant. The conditions of the hydrothermal reaction temperature and time, stirring time, and luminescent properties are analyzed and discussed in detail herein. 

It was demonstrated that a single-crystal nanorod-like YMn_2_O_5_ powder with a uniform size could be obtained by oxidizing the material for 30 min in the air and treating it hydrothermally at 180 °C for 24 h. Full oxidation in the air can change manganese ions from divalent to trivalent and tetravalent. Moreover, the hydrothermal temperature and time are the key factors for determining the nucleation and growth of YMn_2_O_5_. The growth direction of the nanorods was along the crystallography c axis and parallel to the [001] direction. The growth mechanism of YMn_2_O_5_ followed a dissolution–crystallization mechanism. Under the 397 nm excitation, the Mn^4+^ ions exhibited an intense orange emission at 596 nm. The YMn_2_O_5_ powder has potential applications in optoelectronic areas. The energy bandgap of YMn_2_O_5_ powders was 1.18 eV.
